# Supplementary material for: Conditional chemoconnectomics (cCCTomics) as a strategy for efficient and conditional targeting of chemical transmission
Source: eLife. 2024 Apr 30;12:RP91927. doi: 10.7554/eLife.91927 (PMC11060718; doi:10.7554/eLife.91927)
Supplement: Figure 3—source data 3. [file elife-91927-fig3-data3.zip › Figure 3 source data3 part 2.zip!/Figure 3-Source data 3-part2/1025-13-CRISPResso_Report_ack040l3.zip!/CRISPResso_on_1025_15/1a.Read_barplot.pdf]

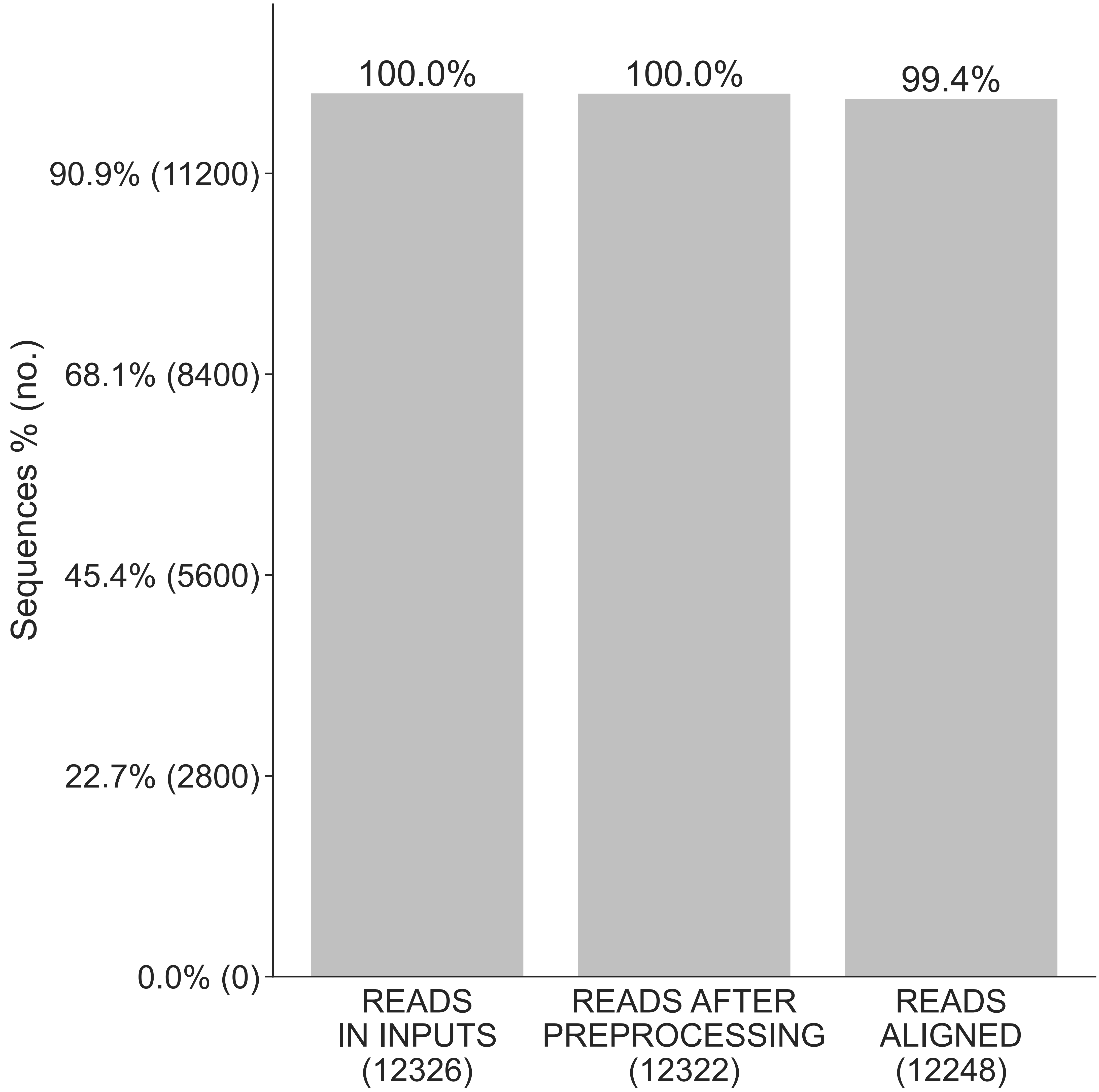

Sequences % (no.)
90.9% (11200)
68.1% (8400)
45.4% (5600)
22.7% (2800)
0.0% (0)
100.0%
100.0%
99.4%
READS IN INPUTS (12326)
READS AFTER PREPROCESSING (12322)
READS ALIGNED (12248)
